# Assessment of feasibility of actigraphy as a measure of clinical change in response to an experimental interventional treatment in adolescents and adults with autism spectrum disorder

**DOI:** 10.3389/fpsyt.2025.1570611

**Published:** 2025-05-23

**Authors:** Matthew Boice, Svetlana Bryant, Matthew Klein, Martine Meyer, Abi Bangerter, Srinivasan Vairavan, Gahan Pandina

**Affiliations:** ^1^ Neuroscience, Janssen Research and Development, Titusville, NJ, United States; ^2^ Department of Psychology, Barker Lab, Rutgers, The State University of New Jersey, Piscataway, NJ, United States

**Keywords:** actigraphy, autism spectrum disorder, biomarker, clinical trials, feasibility, outcome measurement

## Abstract

**Objective:**

The use of actigraphy as a continuous experimental measure of clinical change was explored through a comparison of two clinical studies in autism spectrum disorder (ASD). The data quality, implementation ease, wear compliance, and clinical outcome correlation of actigraphy as a measure were assessed.

**Methods:**

Two clinical studies were conducted and used as a basis of comparison: (1) AUT2001, a Phase 2A interventional study in ASD (N=63), and (2) AUT0002, a Phase 0 non-interventional study in typically-developing (TD) participants (N=53). Participants in both studies wore a wrist-based actigraph throughout enrollment. Actigraphy features were identified based on potential clinical relevance and calculated as weekly averages for each participant’s study timepoints. Expert review was used to confirm validity of automated sleep/wake period detection. Feature differences were then assessed using *t* tests/ANCOVA. Spearman rank correlations between actigraphy features and caregiver reported outcome measures were also examined.

**Results:**

Results from both clinical studies were combined during analysis. Actigraphy was shown to be feasible as a measure of longitudinal change in ASD, but with notable challenges in adherence: participant exclusions due to poor wear compliance substantially reduced the size of the final dataset. Despite this limitation, several findings were noted. Significant differences in sleep disturbance were observed at baseline between the ASD and TD populations as measured by physical activity occurring within the defined sleep period. No significant between-group differences were noted in changes from baseline to endpoint in key sleep variables. Caregiver reported sleep quality significantly correlated with actigraphy measures of sleep disturbance. Additional significant correlations were observed between caregiver reported outcomes of self-regulation and actigraphy features measuring daytime physical activity. Finally, potentially relevant correlations with anxiety, social responsiveness, and restricted and repetitive behaviors are reported.

**Conclusions:**

The observed correlations suggest there may be alignment between some generalized features of actigraphy and core and associated domains of ASD. The clinical utility of actigraphy as a biomarker of clinically relevant outcomes in ASD requires further study. Actigraphy may provide supportive evidence of treatment outcome, providing clinical context, or as a objective behavioral measure (e.g., of sleep or activity level) when combined with more traditional clinical outcome measures.

## 1 Introduction

Digital health technologies (DHTs) have emerged as potential transformative tools to enhance drug development processes. The FDA encourages the implementation of DHTs in clinical trials, highlighting the advantages of remote data collection (1). In particular, wearable devices are a promising approach to investigating the real-world impact of pharmacotherapies and treatment protocols. Low-burden wearable devices are uniquely positioned to facilitate continuous recording of physiological and behavioral data, such as blood pressure, physical activity, and glucose levels with minimal interaction from the user. When integrated into clinical trials, these devices offer cost-effective objective insights into disease physiology and outcomes, breaking through the traditional confines of periodic site visits.

Wearable devices, namely, actigraphy are commonly used for measuring activity using a non-invasive method of measuring motion acceleration over an extended time period. Measures of rest obtained from actigraphy have been more closely associated with output from polysomnography (PSG) than participant-reported sleep diaries (2, 3). Actigraphy has been previously suggested by the American Academy of Sleep Medicine (AASM) as an outcome measure of treatment efficacy (4). Additionally, it is less expensive, less invasive, and more convenient than PSG, as it can be performed at home or in other familiar environments. Moreover, it has the capability to capture the variability and fluctuations of sleep/wake activity rhythms continuously over multiple days, presenting a more representative image of real-life behavior than a single night of PSG (particularly in a sleep lab) or subjectively reported daily observation can provide. In addition to sleep variables, modified activity levels, captured by actigraphy devices are frequently symptoms of neuropsychiatric and neurodegenerative disorders, such as depression and bipolar disorder (5), schizophrenia (6), Alzheimer’s disease (7), and Parkinson’s disease (8).

The ability of actigraphy to capture select behavioral features in a low-burden manner is likely why it has consistently been researched as a relevant source of longitudinal data in autism spectrum disorder (ASD). Pediatric studies in children with ASD indicate sleep disturbance as a prominent feature of the condition (9, 10) which may manifest in several different ways, such as difficulty falling asleep and maintaining sleep, as well as bedtime resistance and early awakening (11). Sleep disturbance may often persist into adulthood – problems with sleep efficiency and sleep onset latency being reported in adults with high-functioning ASD (12). While PSG is the recognized standard for diagnosing sleep disorders, its procedures can be daunting for individuals with ASD due to tactile sensitivity. Moreover, individuals can be negatively affected by a change in routine, and a visit to a sleep center or a hospital, where PSG procedure is commonly performed, can elicit anxiety and discomfort, ultimately affecting procedure itself. Actigraphy represents a practical alternative and has been previously reported to be a valid method to evaluate sleep in the pediatric ASD population, showing good reliability for most sleep parameters (13).

Similarly, differences in waking activity levels between typically developing individuals and children and adults with ASD have been extensively studied (14–18). While reported results have been occasionally contradictory, most have concluded that an observable difference exist and further study is warranted. Beyond generalized activity patterns, individuals with ASD may exhibit stereotypical motor movements, patterns, or aggression that often manifest in a way that actigraphy has the ability to objectively quantify and predict (19, 20). This type of measurement also offers several advantages over standardly used caregiver report outcomes, allowing continuous daily monitoring and eliminating the effect of observer-bias.

In this paper, we present an exploratory analysis that assesses the feasibility and suitability of actigraphy as a measure of clinical change in response to an experimental interventional treatment in adolescents and adults with ASD. We hypothesized that the combination of a continuously worn wearable device and a comprehensive site training, monitoring, and support strategy would enhance wear compliance throughout the study and generate enough data to establish actigraphy as a reliable experimental measure for interventional studies. Furthermore, we predicted that not only would discernible differences emerge between the ASD and typically-developing (TD) populations in actigraphy features of interest, but also that distinguishable variations would be observed between the treatment and placebo groups in response to intervention. Finally, we hypothesized that significant correlations (*P* <.05) would emerge between derived actigraphy features and commonly used caregiver reported outcome measures used in ASD research, and that these correlations would make clinical sense within the context of the feature.

## 2 Materials and methods

### 2.1 Study design and participants

This analysis was part of a larger program that consisted of two clinical trials:

42165279AUT2001: a randomized, multi-center trial (NCT03664232) with the primary objective of assessing the efficacy, safety, and tolerability of JNJ42165279, a highly selective and orally bioavailable fatty acid amide (FAA) hydrolase inhibitor, during 12 weeks of treatment in adolescent and adult subjects with ASD (“AUT2001”) (21). 63 adolescent and adult individuals (between 13 and 35 years of age) with a definitive diagnosis ASD using the Autism Observation Schedule, Second Edition (ADOS-2) and an IQ of ≥60 as measured by the Kaufman Brief Intelligence Test – Second Edition (KBIT-2) were enrolled. Participants were randomized in a 1:1 ratio to receive double-blind treatment of either JNJ42165279 or placebo.

A secondary objective of this trial was to correlate changes in efficacy instruments completed by caregivers with measures of actigraphy. For this analysis we explore a subset of the most clinically relevant instruments used to measure social communication, restrictive/repetitive behaviors, and self-regulation that were thought to have the highest probability of meaningful correlation:

- Autism Behavior Inventory (ABI): 65-item multi-dimensional scale related to the core and associated symptoms of ASD (22, 23). For this analysis, only 3 subscale scores were considered: Restrictive/Repetitive Behaviors, Social Communication, and Self-Regulation. Additionally, the single item related to sleep was considered.
- Social Responsiveness Scale 2 (SRS-2): 5 subscales, 65 items; distinguishes ASD conditions from other child psychiatric conditions by identifying the presence and extent of ASD-specific social impairment; has been used previously as an outcome measure in clinical trials (24).
- Child Adolescent Symptom Inventory – Anxiety (CASI-Anxiety): 21-point anxiety scale which has been suggested as a potential outcome measure in ASD (25). In this analysis, only the CASI-Anxiety Total Score was considered.
- Repetitive Behavior Scale – Revised (RBS-R): a 43-item scale to indicate the occurrence and severity of repetitive behaviors (26). The instrument has been considered previously as a potential outcome measure (27, 28).
- Janssen Autism Knowledge Engine (JAKE) Daily Tracker: a set of questions caregivers were asked to provide daily responses using web/mobile application through a 7-point Likert scale (higher values are considered more positive than lower) (29). In this analysis, 2 items were considered: overall type of day and perceived quality of sleep.

42165279AUT0002: a prospective, non-interventional, multicenter trial in TD participants, serving as a comparison population cohort for the interventional trial (“AUT0002”). 53 TD adolescent and adult individuals (between 13 and 33 years of age) enrolled in the main trial (which consisted of a single site visit); of these participants, 26 took part in a longer observational extension (approximately 4 to 8 weeks). In this case study, we consider only the individuals participating in this extension where sufficient actigraphy data could be collected.

### 2.2 Devices

All participants in both trials received a wrist-wearable ActiGraph™ GT9X Link (510[k]: K080545) accelerometer and were instructed to wear the device at all times (except when charging the device weekly and showering/bathing). To encourage comfort and compliance, participants were permitted to wear the device on either the dominant or non-dominant hand but were instructed to remain consistent with wear position throughout the study. Recorded data was uploaded to the ActiGraph™ CenterPoint system at each site visit (described in **Table 1**). To ensure proper setup and device calibration, study investigators were provided training and access to a dedicated remote support technician that was a member of the study team. Reported issues were actively monitored, reviewed, and resolved by the study team (and in close collaboration with the vendor where necessary).

**Table 1:** Timepoint day ranges in AUT2001 and AUT0002.

Study	Baseline	Day 29 Visit	Day 57 Visit	Endpoint
AUT2001, timepoint range	Day 0 to Day 6	Day 22 to Day 28	Day 50 to Day 56	Day 78 to Day 84
AUT0002, timepoint range	Day 0 to Day 6	–	–	Last Day (-7) to Last Day (-1)

### 2.3 Feature extraction

The triaxial accelerometry data recorded at 30Hz was extracted using ActiGraph™ ActiLife v6.11. The raw time series values were then processed in R using GGIR (v2.7-1) as a convenient, well-documented method to perform autocalibration and initial estimates of sleep and physical activity (30–35 A single activity level classification using cut-points defined by Maria Hildebrand was applied to each participant (light = 46, moderate = 101, vigorous = 429) (36).

**Figure 1:**
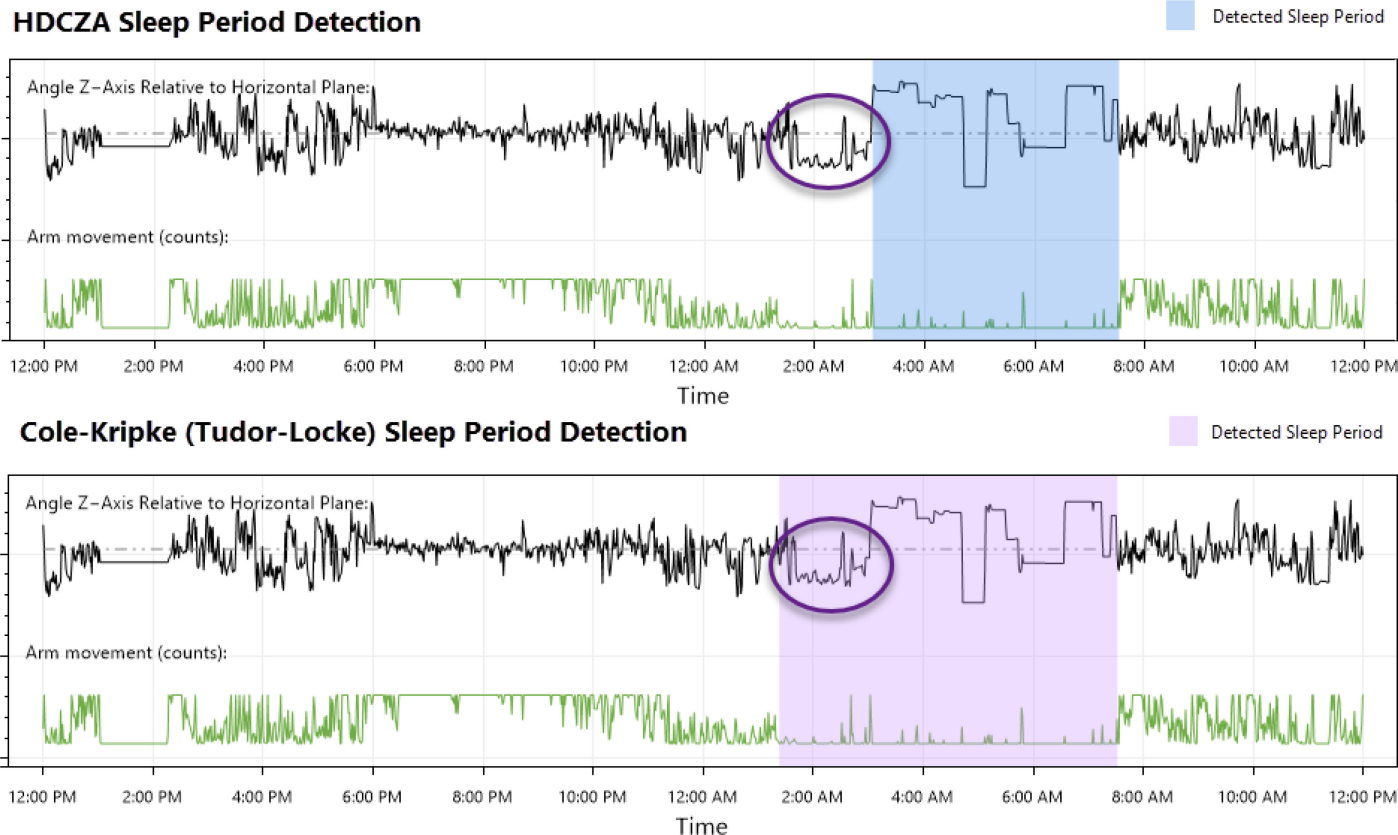
Comparison of an HDCZA detected sleep period and one detected using the Cole-Kripke sleep state algorithm (37) with Tudor-Locke period boundary detection (38), a standard offering available through the ActiGraph™ CentrePoint system.

The sleep estimates were obtained using the HDCZA (Heuristic algorithm looking at Distribution of Change in Z-Angle; 32, 33), a technique that considers device orientation instead of acceleration magnitude to identify wake and sleep states. This approach has several advantages that uniquely position it when attempting to assess individuals with significantly disturbed sleep or extended periods of waking inactivity. The first is the visual distinction it has over the traditionally used ‘counts’ to quantify activity, allowing the opportunity for more meaningful expert review. The second advantage is related to the first: the method allows a qualitative, detailed examination of the movement (estimated Z-axis orientation of the device) instead of a quantitative measure of activity magnitude using counts or ENMO (Euclidean Norm Minus One). This method was particularly useful for identifying sleep period boundaries in individuals with ‘settling time’ prior to sleep period onset (showing qualitative differences between periods of waking inactivity and suspected sleep that would not be apparent if assessing counts or ENMO alone) (**Figure 1**).

**Figure 2:**
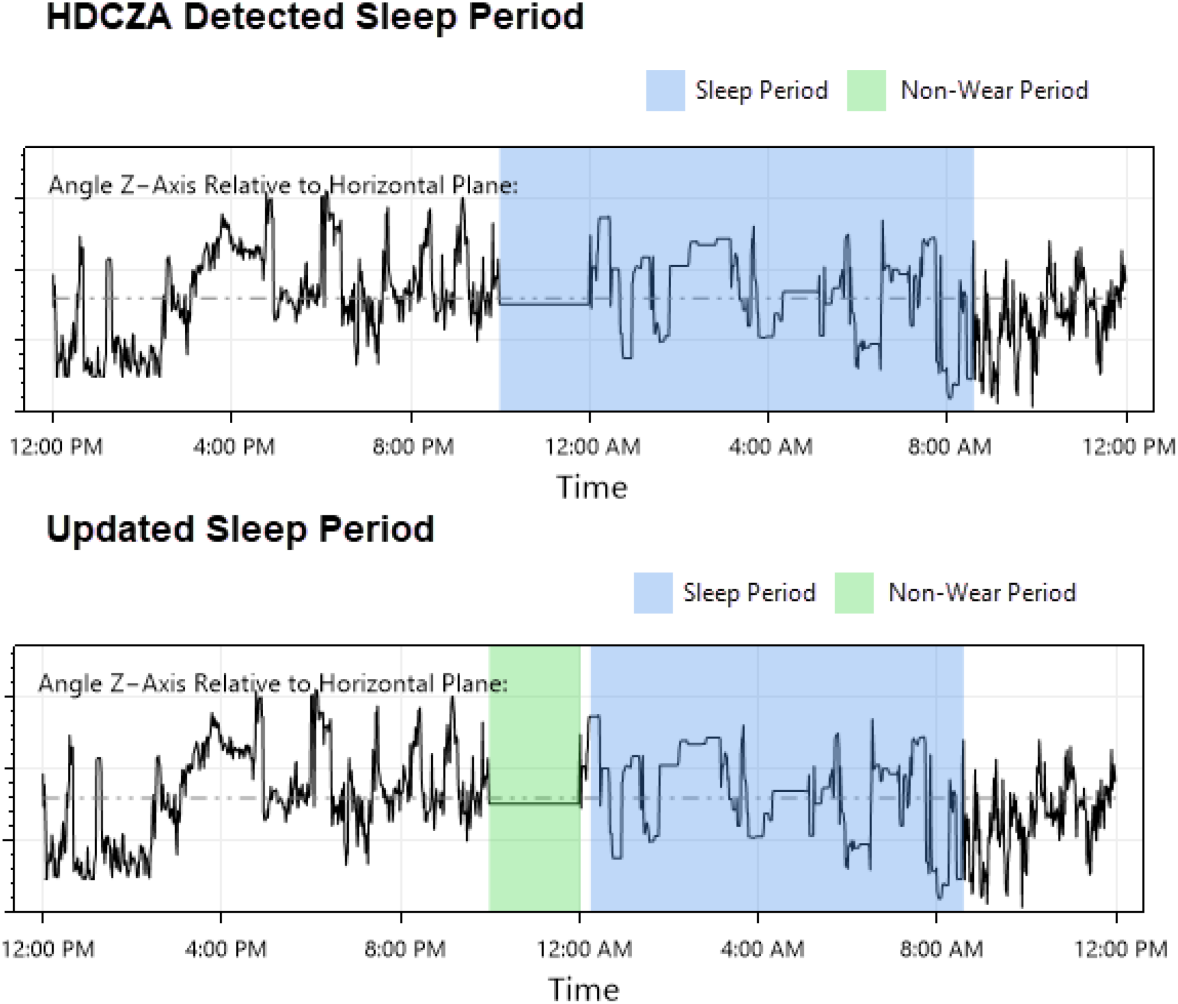
Sleep period detection correction example: a visual inspection/correction procedure was implemented to address issues arising from GGIR’s HDCZA method of detecting sleep and sleep period boundaries: suspected non-wear periods that occurred near a detected sleep period were occasionally combined, resulting in an inaccurate sleep period start time that affected downstream features. The inspector attempted to identify these occurrences, updated the sleep period start time, and provided these values to GGIR for a second run of feature extraction.

Each participant’s data was visually inspected day-by-day to 1.) ensure sleep period detection appeared accurate, and 2.) periods of suspected non-wear were noted for exclusion consideration. In situations where a period was believed to be incorrect (e.g., two sleep periods directly adjacent to each other or periods of suspected non-wear near a sleep period being misidentified as sleep), the inspector adjusted the values using a customized graphical interface (**Figure 2**).

**Figure 3:**
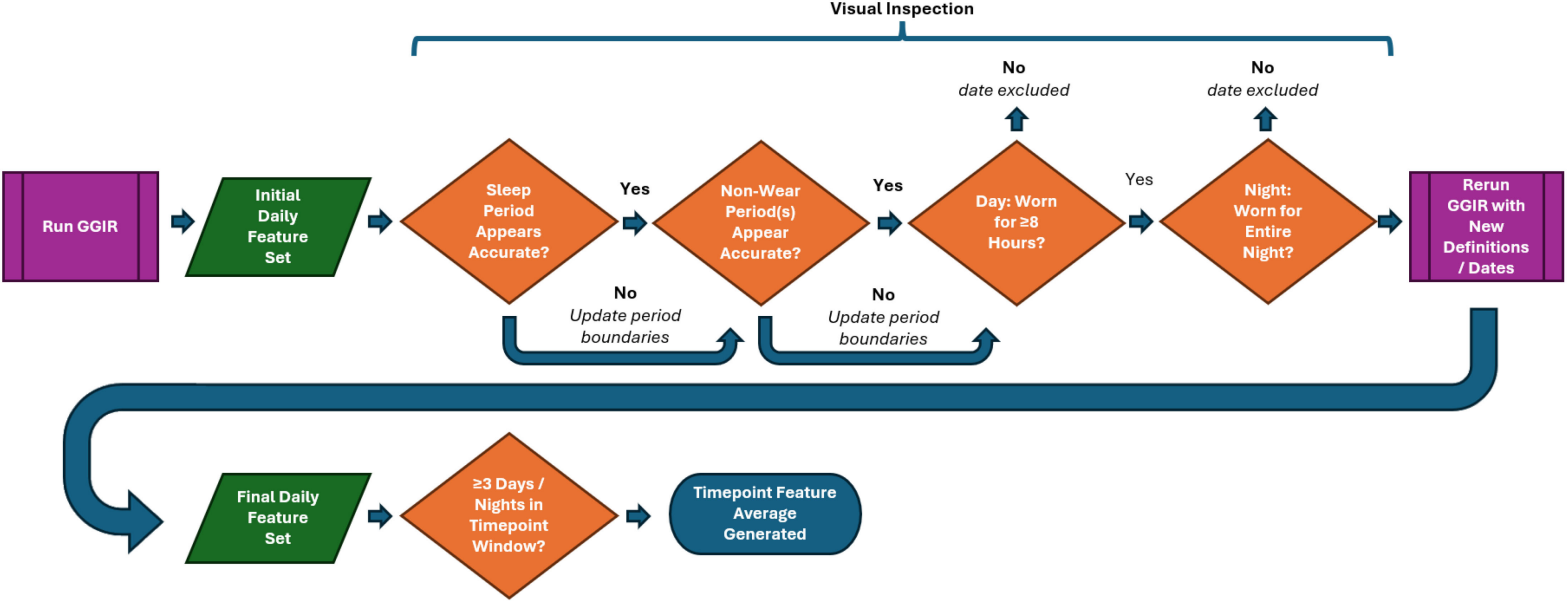
Visual inspection workflow used to determine final feature set used in analyses.

The final period span values were then exported and used as a parameter for a second run of GGIR, yielding a comprehensive set of daily features offered by the package [of which, a subset of 11 were identified/derived as of potential clinical importance (see **Table 2**)]. This complete feature extraction process flow is illustrated in **Figure 3**.

**Table 2:** GGIR actigraphy feature calculations.

Feature	GGIR part5_daysummary Feature Calculation	Description
Duration of MVPA Fragments (mins)	FRAG_mean_dur_MVPA_day	The average duration (in minutes) of detected moderate-to-vigorous physical activity fragments.
Duration of Physical Activity During Sleep Period (mins)	dur_spt_wake_LIG_min + dur_spt_wake_MOD_min + dur_spt_wake_VIG_min	The measured duration of physical activity (light, moderate, vigorous) (in minutes) during the sleep period.
Duration of Sleep During Sleep Period (mins)	dur_spt_sleep_min	The measured duration of sleep (‘sustained inactivity bouts’) (in minutes) during the sleep period.
Duration of Wakeful Inactivity During Sleep Period (mins)	dur_spt_wake_IN_min	The measured duration of physical activity not considered to be sleep or light/moderate/vigorous physical activity during the sleep period.
Number of Blocks of Physical Activity During Sleep Period (#)	Nblocks_spt_wake_LIG + Nblocks_spt_wake_MOD + Nblocks_spt_wake_VIG	The number of total blocks of physical activity (light, moderate, vigorous) that occurred during the sleep period.
Number of Blocks of Sleep During Sleep Period (#)	Nblocks_spt_sleep	The number of total blocks of sleep that occurred during the sleep period.
Number of Blocks of Wakeful Inactivity During Sleep Period (#)	Nblocks_spt_wake_IN	The number of total blocks not considered to be sleep or light/moderate/vigorous physical activity that occurred during the sleep period.
Number of MVPA Fragments (#)	FRAG_Nfrag_MVPA_day	The number of fragments of moderate/vigorous activity that occurred per day (part of the behavioral fragmentation analysis methodology) (39).
Number of Sustained Inactivity Bouts During Wake Period (#)	See “Description”	Derived using GGIR ms3.out list “sib.cla.sum” – the total number of SIBs occurring within the defined waking period and not falling within a period of suspected non-wear.
Duration of Sustained Inactivity Bouts During Wake Period (min)	See “Description”	Derived using GGIR ms3.out list “sib.cla.sum” – the average duration of SIBs in minutes occurring within the defined waking period and not falling within a period of suspected non-wear.
Sleep Efficiency (%)	sleep_efficiency	The percentage of time spent asleep during the entire sleep period.

### 2.4 Statistical analysis

Initial differences between the daily wear percentage values of ASD and TD populations were assessed using *t* test. Analysis of Covariance (ANCOVA) was employed to evaluate any potential effects related to gender, age group (13-17; 18-35), weight, intelligence quotient (“IQ”), wear position (dominant *vs* non-dominant hand) (“WP”), and clinical research site on daily wear percentages:


$$\begin{array}{c} ASD\;Wear\;\%∼TD\;Wear\%+gender+age\;group+IQ+WP\\ +Clinical\;Site \end{array}$$

For all subsequent analyses, daily actigraphy features were first filtered through visual inspection to ensure that derived aggregated timepoint values were as representative as possible: 1.) days that contained less than eight (8) hours of suspected wear were excluded, and 2.) nights where the participant was suspected not to be wearing the device for the entire duration were excluded.

Feature averages were then calculated for each timepoint where ≥ 3 days and nights of non-excluded data were available for each timepoint (i.e., a “valid” timepoint). As further described in **Table 1**, the baseline timepoint range was defined as the participant’s first recording day to the sixth; the endpoint range was defined as the seven days prior to the participant’s last recording day. The ranges for the two mid-study visits that occurred only in AUT2001 (Day 29 and Day 57) were similarly defined as the seven days prior to the planned study visit.

Differences across groups for all characteristics were evaluated using *t* tests. ANCOVA was utilized to examine the potential influence of gender, age group, weight, and IQ on the outcomes. To control false discovery rate (FDR), the Benjamini & Hochberg procedure was implemented using α level of.05 (40).

The following comparisons were examined:


$$\begin{array}{c} ASD\;Baseline\;Average∼TD\;Baseline\;Average+gender+age\;group\\ +weight+IQ+WP \end{array}$$

$$\begin{array}{c} Treatment\;Baseline∼Placebo\;Baseline+gender+age\;group\\ +weight+IQ\\ +WP\;Treatment\;Baseline\;Change∼Placebo\;Baseline\;Change\\ +gender+age\;group+weight+IQ\;+WP \end{array}$$

To be included in ASD ~ TD baseline comparisons (Comparison Set 1), participants required only a valid baseline timepoint. Treatment ~ placebo comparisons (Comparison Set 2) required participants to have both a valid baseline and endpoint.

Additionally, to assess the relatedness of caregiver reported outcomes and actigraphy features, Spearman correlations were calculated between features and completed clinical scales for all ASD population timepoints (baseline, Day 29, Day 57, and endpoint) in Comparison Set 1.

## 3 Results

### 3.1 Demographics

**Table 3** provides the final demographic and data quality details for participants included in the statistical analysis after all filtering steps had been applied. For ASD ~ TD baseline comparisons (ASD [n = 38] and TD [n = 21]), the mean (SD) age of ASD and TD participants were 21.6 years (5.3 years) and 21.8 years (5.63 years), respectively. For all comparisons evaluating change over time (treatment [n = 10] and placebo [n = 17]), the mean (SD) age of the treatment and placebo were 22.7 years (7.21 years) and 21.12 years (4.15 years), respectively.

**Table 3:** Final demographic and data quality statistics.

Population	Original		With Valid Actigraph Data	With ≥ 3 days per required timepoint(s)		
ASD (baseline), N (%)	63		38 (60%)	35 (56%)		
TD (baseline), N (%)	26		21 (81%)	20 (77%)		
Tx (baseline/endpoint), N (%)	32		16 (50%)	10 (31%)		
PBO (baseline/endpoint), N (%)	31		22 (71%)	17 (55%)		
Description	Comparison Set 1		Comparison Set 2			
	ASD (N = 35)	TD (N = 20)	Tx (N = 10)		PBO (N = 17)	
Days per timepoint, average (SD)	6.54 (0.82)	6.45 (1.05)	6.9 (0.32)		6.71 (0.59)	
Days per timepoint, median (range)	7 (4 - 7)	7 (3 - 7)	7 (6 - 7)		7 (5 - 7)	
Daily wear %, average (SD)	87.26 (15.78)	88.22 (8.56)	95.22 (2.51)		92.8 (4.99)	
Daily wear %, median (range)	93.58 (32 - 99.15)	91.74 (72.23 - 97.33)	94.92 (91.96 - 98.95)		94.3 (83.85 - 99.15)	
Age (years), average (SD)	21.6 (5.3)	21.8 (5.63)	22.7 (7.21)		21.12 (4.15)	
Age (years), median (range)	21 (14 - 36)	22.5 (13 - 32)	20.5 (16 - 36)		21 (14 - 29)	
13 to 17 years, N (%)	8 (22.86%)	5 (25%)	4 (40%)		2 (11.76%)	
Male, N (%)	28 (80%)	14 (70%)	7 (70%)		14 (82.35%)	
Weight (kg), average (SD)	76.98 (21.13)	75.49 (18.27)	87.08 (27.18)		72.22 (15.16)	
Weight (kg), median (range)	75.53 (40.09 - 122.18)	75.28 (41.72 - 108.84)	93.08 (40.09 - 122.18)		66.76 (47.62 - 98.82)	
IQ; average (SD)	97.44 (16.03)	107.25 (12.1)	103.8 (13.33)		91.41 (13.64)	
IQ; median (range)	95.5 (63 - 125)	106 (86 - 136)	108 (86 - 119)		91 (69 - 118)	
Dominant Hand WP; N (%)	5 (14.29%)	5 (25%)	1 (10%)		3 (17.65%)	

### 3.2 Data quality

As noted above, **Table 3** provides a summary of the final data set determined to be usable from a quality perspective. For Comparison Set 1, of the original 63 participants in the ASD group, only 35 (56%) had a baseline timepoint that met the minimum requirement of ≥ 3 days/nights. The TD had a higher percentage of valid data: of the original 26 participants, 20 (77%) had a valid baseline timepoint for comparison. Both ASD and TD groups had similar daily wear compliance metrics, with a daily wear percentage of approximately 87% (*non-wear minutes/1440*) and an average of 6 non-excluded days per timepoint.

For participants in Comparison Set 2 (which required both valid baseline and endpoint timepoints), the amount of usable data was significantly less. Treatment performed significantly worse than placebo: of the original 32 participants, only 10 (31%) participants in the treatment group had both timepoints versus 17 (61%) of 31 in the placebo group. Daily wear compliance was roughly similar in both groups, with an average daily wear percentage and days per timepoint value across the groups of 93% and 6, respectively.

Using daily wear percentage as a general estimate of wear adherence, *t* test and ANCOVA analyses showed no significant differences between the ASD and TD populations (*P* = .68). However, some significant effects related to IQ were observed (higher IQ correlated with lower daily wear percentage) (described in **Table 4**).

**Table 4:** Daily wear statistics for all participants in AUT2001 and AUT0002.

Daily Wear Percentages by Group	
ASD (N = 38), average wear percentage	87.72%
TD (N = 21), average wear percentage	86.86%
Tx (N = 16), average wear percentage	85.8%
PBO (N = 22), average wear percentage	89.07%
ASD | TD Daily Wear % Comparisons (*P* = .680)	
Gender	*P* = .740
Age Group	*P* = .740
Weight	*P* = .821
IQ	*P* = .002
Dominant Hand WP	*P* = .782
Clinical Site	*P* = .094

A summary of issues related to actigraphy data collection is presented in **Table 5**. During the study, 67 actigraphy-related support issues were reported. More than half of the reported issues were related to clinical personnel system access, software update assistance, or general question-and-answers (37, 55%) and were frequently resolved within the same day with no impact on data collection. Approximately 7 “halt” issues occurred, where device recording would halt due to a critically low battery level. In all cases, this was attributable to extended periods (> 1 week) of participant non-compliance.

**Table 5:** Summary of actigraphy support issues reported during AUT2001 and AUT0002.

Category	Description	Total
Dashboard Access	Issues related to ActiGraph™ CentrePoint system access for clinical site personnel (account provisioning, forgotten passwords)	18
Software Updates	Requests to assist with updates to the ActiGraph™ ActiSync application	15
Training-Related	Issues related to clinical site personnel requiring additional training	10
Device Halt Issues	Issues related to participants letting the device battery drain to critical levels (often resulting in data loss)	7
Hardware Issue (Device)	Failures related to the ActiGraph™ GT9X Link device (broken devices/wristbands, data transfer errors)	7
Q&A	Requests for additional information from clinical site personnel	4
Server Error/Outage	Issues related to ActiGraph™ CentrePoint system outages	3
Network Connectivity	Issues related to clinical site network connectivity access	2
Lost Device	Occurrences of participant losing their device	1

### 3.3 ASD/TD comparison set 1

We compared differences for each of the baseline averages of the identified features of interest between the ASD and TD participants in Comparison Set 1. As shown in **Table 6**, only 2 features were found to be significant: “Duration of Physical Activity During Sleep Period” and “Number of Blocks of Physical Activity During Sleep Period” (*P* = .007 | Cohen’s *d* > 0.8) – with ASD experiencing greater durations and discrete occurrences of physical activity during the sleep period than TD. Furthermore, **Table 7** shows that there does not appear to be any significant difference regarding gender, age group, weight, IQ, or wear position for either feature (*P* >.5).

**Table 6:** t test results summary: *P* (Cohen’s *d*).

Feature	ASD ~ TD Baseline	Tx ~ PBO Baseline	Tx ~ PBO Change from Baseline
Duration of MVPA Fragments (mins)	.796 (0.06)	.313 (0.47)	.221 (0.62)
Duration of Physical Activity During Sleep Period (mins)	.007 (0.83)	.053 (0.94)	.946 (0.03)
Duration of Sleep During Sleep Period (mins)	.363 (0.32)	.484 (0.31)	.132 (0.88)
Duration of Wakeful Inactivity During Sleep Period (mins)	.379 (0.31)	.074 (0.94)	.163 (0.88)
Number of Blocks of Physical Activity During Sleep Period (#)	.007 (0.8)	.084 (0.73)	.516 (0.3)
Number of Blocks of Sleep During Sleep Period (#)	.52 (0.24)	.074 (0.86)	.132 (0.98)
Number of Blocks of Wakeful Inactivity During Sleep Period (#)	.34 (0.43)	.04 (1.06)	.132 (1.16)
Number of MVPA Fragments (#)	.131 (0.51)	.313 (0.43)	.516 (0.26)
Number of Sustained Inactivity Bouts During Wake Period (#)	.758 (0.11)	.026 (1.26)	.163 (0.89)
Duration of Sustained Inactivity Bouts During Wake Period (min)	.34 (0.44)	.04 (0.95)	.384 (0.37)
Sleep Efficiency (%)	.363 (0.33)	.074 (0.84)	.324 (0.61)

**Table 7:** ANCOVA results summary: *P*.

Feature	ASD ~ TD Gender	ASD ~ TD Age Group	ASD ~ TD Weight	ASD ~ TD IQ	ASD ~ TD WP
Duration of MVPA Fragments (mins)	.707	.981	.707	.619	.981
Duration of Physical Activity During Sleep Period (mins)	.92	.801	.707	.65	.981
Duration of Sleep During Sleep Period (mins)	.144	.608	.652	.526	.981
Duration of Wakeful Inactivity During Sleep Period (mins)	.38	.088	.707	.526	.981
Number of Blocks of Physical Activity During Sleep Period (#)	.961	.981	.866	.619	.981
Number of Blocks of Sleep During Sleep Period (#)	.264	.026	.652	.526	.981
Number of Blocks of Wakeful Inactivity During Sleep Period (#)	.38	.077	.652	.526	.981
Number of MVPA Fragments (#)	.92	.624	.707	.619	.981
Number of Sustained Inactivity Bouts During Wake Period (#)	.264	.608	.652	.619	.981
Duration of Sustained Inactivity Bouts During Wake Period (min)	.078	.457	.707	.762	.981
Sleep Efficiency (%)	.314	.417	.83	.526	.981

### 3.4 Treatment/placebo comparison set 2

Several significant differences were observed between the treatment and placebo groups at baseline: “Number of Blocks of Wakeful Inactivity During Sleep Period” (*P* = .04 | Cohen’s *d* > 1.06), “Number of Sustained Inactivity Bouts During Wake Period” (*P* = .026 | Cohen’s *d* > 1.26), and “Duration of Sustained Inactivity Bouts During Wake Period” (*P* = .04 | Cohen’s *d* > 0.95). Additional potentially significant differences were noted as well, with uncorrected *P* values ≤.05 and/or large effect sizes (Cohen’s *d* ≥ 0.8).

No significant differences between the treatment and placebo groups in relation to change from the baseline average were observed in any actigraphy feature, but several were noted as potentially significant prior to FDR correction.

We include all relevant uncorrected *P* values in the **Supplementary Materials** of this paper.

### 3.5 Relationship between actigraphy and caregiver reported outcomes

As shown in **Table 8**, there were 3 caregiver reported outcomes that exhibited significant correlation with actigraphy features (*P* <.05): ABI Self-Regulation, ABI Sleep Item, and SRS-2 Total Score.

**Table 8:** Spearman rank correlations of caregiver reported outcomes and actigraphy features (|ρ| ≤ 0.3).

Caregiver Reported Outcome	Feature	ρ (*P*)
ABI Self-Regulation	Number of MVPA Fragments (#)	0.349 (.007)
	Duration of Sleep During Sleep Period (mins)	0.319 (.007)
	Number of Sustained Inactivity Bouts During Wake Period (#)	0.311 (.009)
ABI Sleep Item	Duration of Physical Activity During Sleep Period (mins)	0.318 (.009)
SRS-2 Total Score	Duration of Sleep During Sleep Period (mins)	0.385 (.007)

**Figure 4:**
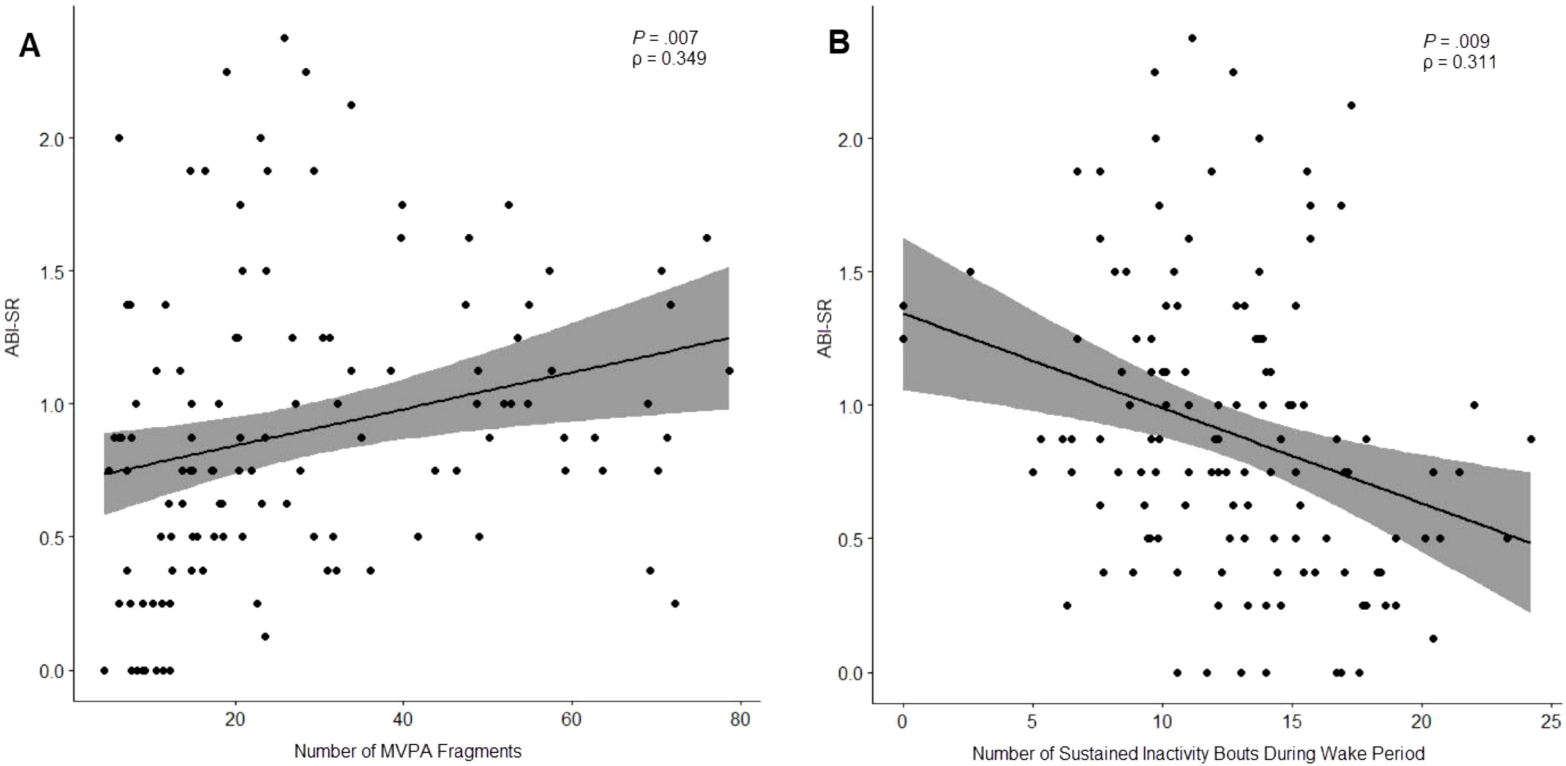
Relationship between ABI Self-Regulation subdomain and number of moderate-or-vigorous activity (MVPA) fragments **(A)** and number of sustained inactivity bouts (SIBs) during waking hours **(B)**.

Discrete occurrences of moderate-or-vigorous activity (MVPA) were found to be significantly correlated to the ABI Self-Regulation subdomain: a higher average number of MVPA occurrences was correlated with worse clinical outcomes (ρ = 0.349, *P* = .007) while the number of discrete occurrences of sedentary activity during waking hours (“Number of Sustained Inactivity Bouts During Wake Period”) was negatively correlated (ρ = -0.311, *P* = .009), suggesting a relationship between waking activity patterns and clinical outcomes of self-regulation (**Figure 4**).

“Duration of Physical Activity During Sleep Period” was noted to have a modest correlation with the ABI Sleep Item (ρ = -0.318, *P* = .009), showing higher levels of physical activity were associated with worse outcomes on this clinical measure of sleep disturbance.

**Figure 5:**
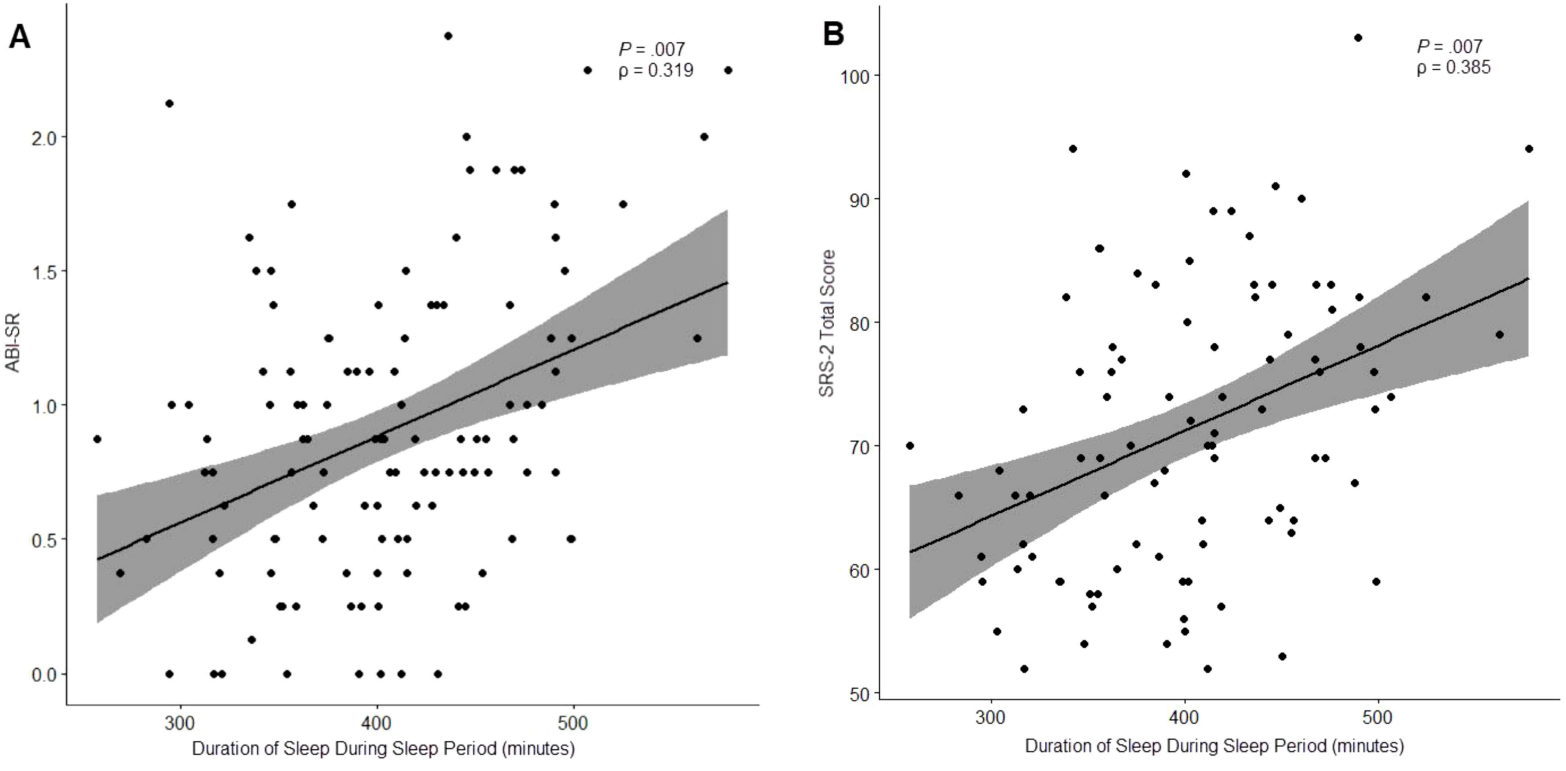
Relationship between “Duration of Sleep During Sleep Period” and ABI Self-Regulation **(A)** and the SRS-2 Total Score **(B)**.

Finally, “Duration of Sleep During Sleep Period” was significantly correlated with 2 clinical outcomes: ABI Self-Regulation (ρ = 0.319, *P* = .007), and SRS-2 Total Score (ρ = 0.385, *P* = .007). Notably, both observed correlations suggested that longer durations of sleep during the sleep period were associated with worse outcomes (**Figure 5**).

Other potentially significant correlations were observed prior to FDR correction. These uncorrected values are included in the **Supplementary Materials** of this paper.

## 4 Discussion & key findings

Research in ASD to date has consistently focused on pediatric populations (41, 42); and while the field is beginning to devote additional attention to research across the lifespan in ASD, longitudinal biometric data sets of non-pediatric autistic populations remain scant. We present our observations below to encourage further exploration in these areas but acknowledge that no definitive clinical conclusions should be reached without larger, controlled population samples.

### 4.1 Wear compliance

Wear compliance greatly reduced the data set used in the final analyses, with a significant impact on the statistical power of the results and interpretability of conclusions. To make any valid statistical inferences of change over time within comparative populations, a baseline and endpoint of sufficient size for each participant is necessary. In this study, the largest impact on the final sample size of Comparison Set 2 was due to missing endpoint visits. The dropout rate was similar between the two groups: 6 participants from treatment were excluded, and 4 from the placebo. In most cases, the exclusions were attributed to lack of daily wear compliance (< 8 hours per day) or failure to wear the device to bed within the 3-day endpoint window.

Exclusions were not confined to missing endpoints: 4 participants were missing a baseline visit (3 in treatment, 1 in the placebo). Of these, 2 were attributed to a site-training issue, 1 was related to a technical issue (corruption of the recording file), and 1 was related to poor compliance during baseline (having only a single non-excluded day during the timepoint period).

Another confounding factor was the non-wearing of the device by a subset of participants during sleep, with 7 participants having greater than 40% of nights excluded due to non-wear. As 6 of 10 actigraphy features relied upon valid wear during sleep, consistent night-time wear was critical. All 7 participants were excluded from the treatment/placebo analyses.

However, the remaining sample (treatment [N = 10], placebo [N = 17]) demonstrated exceptional compliance with daily wear requirements (both groups exceeding the number of required days per timepoint and averaging a daily wear percentage of approximately 93% or 22 hours per day). Furthermore, wear compliance was largely consistent across demographic considerations, with IQ being the sole outlier: lower scores correlated with higher wear compliance percentages. This may suggest a challenge to our typical assumptions about conducting studies in lower functioning populations. Research has frequently shied away from more severely affected populations due to the complexity it may introduce (43). These results suggest that with proper participant training, guidance, and desensitization, actigraphy can be effective, feasible measure within lower functioning individuals.

### 4.2 Device considerations

At the time of study design in 2018, the ActiGraph™ GT9X Link was selected for its balance of several key features. The design was simple, unobtrusive, and allowed for raw actigraphy data capture at a high sampling rate without any significant compromise to battery life. Additionally, device storage capacity allowed for longer periods of continuous wear without needing to implement a remote data uploading strategy. However, while the latter eased implementation logistics and reduced costs, it also impacted the ability to monitor wear compliance on a more frequent basis and reduced the opportunity for site personnel to intervene early.

Limitations of the device were evident when attempting to accurately measure sleep onset, waking, and sedentary behavior during waking hours. While the ActiGraph™ GT9X Link includes a capacitive proximity sensor to assist with wear-time detection, findings suggest it is frequently unable to reliably distinguish between states (44). Furthermore, as the device does not include a skin temperature, EDA (electrodermal activity), or PPG (photoplethysmogram) sensor, it was necessary to rely solely upon raw accelerometry to determine wear state. An automated non-wear detection method natively available through GGIR 2.7-1 (45, 46), was implemented initially, but visual inspection of the data revealed issues with this approach in a free-living study population.

It was observed that GGIR’s HDCZA method of sleep detection would occasionally misestimate the sleep period start time if it occurred near a detected non-wear period. The reverse was also observed: final wake-up timing could be delayed if a period of suspected non-wear was close by. This was confirmed to impact the final sleep features generated by the GGIR. To accommodate this, we developed a graphical user interface to adjust the detected sleep periods through expert review. These adjusted sleep period definitions were then passed to GGIR using its “sleeplog” parameter and used to create the final feature set.

Similarly, the GGIR’s non-wear detection would also occasionally fail to detect periods of non-wear if any acceleration was detected during the period. These periods would often be visually inconsistent with other periods of low activity in the same participant at high resolutions (1 second).

Due to this observed lack of specificity, we implemented a similar visual inspection/adjustment procedure for detected non-wear periods (and these form the basis of the wear percentage values we report in this study). Features measuring sedentary behavior during waking hours (“Number of Sustained Inactivity Bouts During Wake Period” and “Duration of Sustained Inactivity Bouts During Wake Period”) accounted for these adjustments.

It is important to note that more recent versions of GGIR have released adjusted methods for non-wear detection which could potentially alleviate these issues. However, our observations and experience would indicate that a robust, scalable wear-detection strategy should be multi-modal, utilizing a combination of information from accelerometer, temperature, EDA, and/or PPG sensors. An accurate wear-detection procedure is necessary to confidently measure periods of low activity during waking hours, a measure generally of interest and importance across many disorders, and critical when considering measures of behavioral rhythmicity over long periods of time.

### 4.3 Analysis and interpretation challenges

Given that measures of sleep disturbance have consistently been found to relate to the core and associated symptoms of ASD (47–51), the use of actigraphy to measure sleep in individuals, particularly children, with ASD is not novel. However, the techniques of past studies have frequently lacked standardization, with variations in device manufacturer, data collection duration, epoch length, scoring algorithms, and rules for handling missing data. While open-source data analysis tools for actigraphy are available in programming languages such as R and Python, studies often adopt divergent methodologies for data analysis and are frequently not validated within the population of study.

Furthermore, automated sleep period detection in actigraphy presents real challenges in individuals with severely disturbed sleep or sedentary lifestyles. Visual review of the data frequently revealed sleep periods that were often interrupted by several minutes of light, moderate, or vigorous physical activity multiple times, resulting in more than one automatically detected period per night. This presents an analysis problem: to consider such segments of activity as awakening events and recombine the detected periods, or to consider each separately and average the values? The latter approach may potentially result in an inaccurate depiction of a participant’s sleep (for example, skewing measures of sleep efficiency). In this analysis, the former approach was adopted, but that too carried its own problem: how long of an awakening event is too long before the periods should be considered separately? This is a question worth further consideration, especially when evaluating participants with serious sleep problems.

Also noteworthy was the emergence of correlation between increased sleep duration and worse clinical outcomes on the ABI Self-Regulation and SRS-2 scores. To a lesser extent, the same relationship was observed in four other outcomes prior to FDR corrections (as reported in the **Supplementary Materials**). No explanation when examining measures of sleep disturbance or sleep efficiency was found in this analysis: no features of sleep disturbance demonstrated any correlation with caregiver outcomes associated with the core symptoms of ASD (before or after FDR correction); paradoxically, higher sleep efficiency was correlated with worse outcomes in self-regulation (prior to FDR correction). These observations appear to challenge past findings and assumptions. This could simply be an artifact resulting from the insufficiently powered sample, or potentially due to the suspected imbalance of sleep disturbance incidence between the treatment and placebo populations (as suggested by the large effect sizes in all related features observed between the treatment and placebo at baseline). However, it could also be the result of the HDCZA method itself, not being specifically validated within ASD – measures of sleep duration may not actually be measures of restorative sleep but simply a state of motionlessness. Finally, it may be that the relationship between sleep disturbance and the core symptoms of ASD is not perfectly linear or universal. If the opposite were true, clinical practice would likely place more focus on treating sleep disorders to elicit improvement in core symptoms.

Finally, worth further consideration are our findings that showed a relationship between self-regulation and generalized features of daily waking. This relationship demonstrates some clinical validity: increased levels of MVPA correlating with worse clinical outcomes while more frequent and longer sustained periods of rest correlating with better ones. This suggests that these types of generalized actigraphy measures may be useful in building a picture of symptom burden. Still, it is important to remember they are not measuring the same construct as standard clinical instruments and should not be considered as analogs. ASD is not a movement disorder; and while many neurodevelopmental/neuropsychiatric disorders may manifest physically, the presentation may not always be consistent (e.g., ‘anxiety’ may result in an increase or decrease of physical behavior depending on learned coping strategies). Indeed, our results showed that these features did not significantly discriminate between ASD and TD populations. As such, a precision approach may be useful when considering generalized actigraphy features, considering absolute change from an individual’s baseline or defining a population subtype, but this concept is still novel in the world of clinical trials. Alternatively, actigraphy may be considered in situations where consistent relationships with physical behavior exist (for example, the linkage between depressive symptoms and sedentary behavior). However, measurement of activity does not tell us alone about a participant’s experience (“Am I feeling better or am I just moving more?”). It seems unlikely that actigraphy will have the same clinical relevance as traditional gold-standard outcome measures when considering purely psychiatric symptoms. However, it may be useful as an objective, supportive end point [providing additional validation for a primary, symptom-specific endpoint (“I’m feeling better and I’m moving more”)].

## 5 Conclusions

Operationalization of actigraphy on a large scale remains a challenge, and while many of the problems are complex and often involve external factors outside of the study team’s control, they are not insurmountable. Mitigating strategies might include a screening period to assess a participant’s likelihood of remaining compliant throughout the study’s duration or more frequent, active monitoring by local site personnel. Regular communication with participants could identify and alleviate individual challenges that often lead to poor compliance, such as problems with comfort (especially during sleep). Additionally, considering alternative wear positions (e.g., waist, shoe) may be optimized for the study population, though it is important to note that these positions will also dictate the types of features that can be collected and the degree of validation. Study designs may also need to factor in a certain percentage of participants that will be non-compliant with wear requirements regardless of all attempts at guidance or intervention when determining the appropriate sample size or consider a more flexible approach to timepoint analysis (e.g., considering an endpoint within a sliding window).

Still more complicated are problems that may arise with clinical interpretation. Any definitive interpretation requires a sufficiently powered sample set, which proved to be a challenge in this analysis. Moreover, noted baseline differences between the treatment and placebo groups further challenge our ability draw truly meaningful clinical conclusions. Finally, movement is highly individualized, and not all devices or analytical strategies may be equal (or appropriate) for all situations or all neurodevelopmental/neuropsychiatric disorders. As devices improve (with the introduction of larger batteries and more varied, higher precision sensor arrays), some of these problems may be alleviated. Considering the above, current evidence suggests that actigraphy may provide supportive evidence of treatment efficacy when used alongside traditional ASD outcome measures; however, for it to serve as a reliable, clinically relevant measure of change, further development of devices and enhancements in implementation and analysis strategies may be necessary.

## Data Availability

The datasets presented in this article are not readily available because these data are wholly owned by Johnson & Johnson Innovative Medicine, and are subject to company policies. Requests to access the datasets should be directed to gpandina@its.jnj.com.
