# Supplementary material for: Assessment of feasibility of actigraphy as a measure of clinical change in response to an experimental interventional treatment in adolescents and adults with autism spectrum disorder
Source: Front Psychiatry. 2025 May 23;16:1570611. doi: 10.3389/fpsyt.2025.1570611 (PMC12143325; doi:10.3389/fpsyt.2025.1570611)
Supplement: Supplementary file 1 [file Table1.docx]

Table 1. *t* test results summary (uncorrected): *P*

| Feature | ASD ~ TD Baseline | Tx ~ PBO Baseline | Tx ~ PBO Change from Baseline |
| --- | --- | --- | --- |
| Duration of MVPA Fragments (mins) | .796 | .285 | .121 |
| Duration of Physical Activity During Sleep Period (mins) | .001 | .019 | .946 |
| Duration of Sleep During Sleep Period (mins) | .209 | .484 | .029 |
| Duration of Wakeful Inactivity During Sleep Period (mins) | .276 | .041 | .074 |
| Number of Blocks of Physical Activity During Sleep Period (#) | .001 | .061 | .469 |
| Number of Blocks of Sleep During Sleep Period (#) | .425 | .043 | .036 |
| Number of Blocks of Wakeful Inactivity During Sleep Period (#) | .14 | .008 | .014 |
| Number of MVPA Fragments (#) | .036 | .264 | .446 |
| Number of Sustained Inactivity Bouts During Wake Period (#) | .689 | .002 | .059 |
| Duration of Sustained Inactivity Bouts During Wake Period (min) | .154 | .011 | .279 |
| Sleep Efficiency (%) | .231 | .047 | .206 |
