# Supplementary material for: Assessment of feasibility of actigraphy as a measure of clinical change in response to an experimental interventional treatment in adolescents and adults with autism spectrum disorder
Source: Front Psychiatry. 2025 May 23;16:1570611. doi: 10.3389/fpsyt.2025.1570611 (PMC12143325; doi:10.3389/fpsyt.2025.1570611)
Supplement: Supplementary file 2 [file Table2.docx]

Table 2. ANCOVA results summary (uncorrected): *P*

| Feature | ASD ~ TD Gender | ASD ~ TD Age Group | ASD ~ TD Weight | ASD ~ TD IQ | ASD ~ TD WP |
| --- | --- | --- | --- | --- | --- |
| Duration of MVPA Fragments (mins) | .514 | .981 | .355 | .507 | .469 |
| Duration of Physical Activity During Sleep Period (mins) | .782 | .656 | .501 | .591 | .553 |
| Duration of Sleep During Sleep Period (mins) | .026 | .387 | .237 | .147 | .614 |
| Duration of Wakeful Inactivity During Sleep Period (mins) | .242 | .024 | .527 | .227 | .838 |
| Number of Blocks of Physical Activity During Sleep Period (#) | .961 | .905 | .866 | .404 | .439 |
| Number of Blocks of Sleep During Sleep Period (#) | .096 | .002 | .111 | .134 | .969 |
| Number of Blocks of Wakeful Inactivity During Sleep Period (#) | .217 | .014 | .237 | .239 | .899 |
| Number of MVPA Fragments (#) | .836 | .454 | .578 | .49 | .094 |
| Number of Sustained Inactivity Bouts During Wake Period (#) | .094 | .381 | .132 | .413 | .981 |
| Duration of Sustained Inactivity Bouts During Wake Period (min) | .007 | .208 | .545 | .762 | .822 |
| Sleep Efficiency (%) | .143 | .152 | .755 | .22 | .674 |
