# Supplementary material for: Assessment of feasibility of actigraphy as a measure of clinical change in response to an experimental interventional treatment in adolescents and adults with autism spectrum disorder
Source: Front Psychiatry. 2025 May 23;16:1570611. doi: 10.3389/fpsyt.2025.1570611 (PMC12143325; doi:10.3389/fpsyt.2025.1570611)
Supplement: Supplementary file 3 [file Table3.docx]

Table 3. Spearman rank correlations of caregiver reported outcomes and actigraphy features (uncorrected) (*P* < .05)

| Caregiver Reported Outcome | Feature | ρ (*P*) |
| --- | --- | --- |
| ABI Restrictive / Repetitive Behaviors | Duration of Sleep During Sleep Period (mins) | 0.224 (.015) |
|  | Number of MVPA Fragments (#) | 0.219 (.017) |
| ABI Self-Regulation | Duration of MVPA Fragments (mins) | -0.211 (.022) |
|  | Duration of Sleep During Sleep Period (mins) | 0.319 (< .001) |
|  | Duration of Sustained Inactivity Bouts During Wake Period (min) | 0.249 (.005) |
|  | Number of MVPA Fragments (#) | 0.349 (< .001) |
|  | Number of Sustained Inactivity Bouts During Wake Period (#) | -0.311 (< .001) |
|  | Sleep Efficiency (%) | 0.203 (.028) |
| ABI Sleep Item | Duration of Physical Activity During Sleep Period (mins) | 0.318 (< .001) |
|  | Number of Blocks of Physical Activity During Sleep Period (#) | 0.27 (.003) |
| CASI-Anxiety Total Score | Duration of Sleep During Sleep Period (mins) | 0.288 (.005) |
| Overall Type of Day | Duration of Sleep During Sleep Period (mins) | -0.19 (.043) |
| RBS-R Total Score | Duration of Sleep During Sleep Period (mins) | 0.288 (.005) |
| SRS-2 Total Score | Duration of Sleep During Sleep Period (mins) | 0.385 (< .001) |
|  | Duration of Sustained Inactivity Bouts During Wake Period (min) | 0.258 (.011) |
|  | Number of Sustained Inactivity Bouts During Wake Period (#) | -0.24 (.018) |
